# Mismatch repair protein mutations in isocitrate dehydrogenase (IDH)-mutant astrocytoma and IDH-wild-type glioblastoma

**DOI:** 10.1093/noajnl/vdad085

**Published:** 2023-07-12

**Authors:** Timothy E Richardson, Raquel T Yokoda, Omid Rashidipour, Meenakshi Vij, Matija Snuderl, Steven Brem, Kimmo J Hatanpaa, Samuel K McBrayer, Kalil G Abdullah, Melissa Umphlett, Jamie M Walker, Nadejda M Tsankova

**Affiliations:** Department of Pathology, Molecular and Cell-Based Medicine, Icahn School of Medicine at Mount Sinai, New York, New York, USA; Department of Pathology, Molecular and Cell-Based Medicine, Icahn School of Medicine at Mount Sinai, New York, New York, USA; Department of Pathology, Molecular and Cell-Based Medicine, Icahn School of Medicine at Mount Sinai, New York, New York, USA; Department of Pathology, Molecular and Cell-Based Medicine, Icahn School of Medicine at Mount Sinai, New York, New York, USA; Department of Pathology, New York University Langone Health, New York, New York, USA; Department of Neurosurgery, Perelman School of Medicine, University of Pennsylvania, Philadelphia, Pennsylvania, USA; Department of Pathology, University of Texas Southwestern Medical Center, Dallas, Texas, USA; Simmons Comprehensive Cancer Center, University of Texas Southwestern Medical Center, Dallas, Texas, USA; Children’s Medical Center Research Institute, University of Texas Southwestern Medical Center, Dallas, Texas, USA; Department of Neurosurgery, University of Pittsburgh School of Medicine, Pittsburgh, Pennsylvania, USA; Department of Pathology, Molecular and Cell-Based Medicine, Icahn School of Medicine at Mount Sinai, New York, New York, USA; Department of Pathology, Molecular and Cell-Based Medicine, Icahn School of Medicine at Mount Sinai, New York, New York, USA; Nash Family Department of Neuroscience, Icahn School of Medicine at Mount Sinai, New York, New York, USA; Department of Pathology, Molecular and Cell-Based Medicine, Icahn School of Medicine at Mount Sinai, New York, New York, USA; Nash Family Department of Neuroscience, Icahn School of Medicine at Mount Sinai, New York, New York, USA

**Keywords:** astrocytoma, chromosomal instability, glioblastoma, genomic instability, mismatch repair deficit

## Abstract

**Background:**

Mutations in mismatch repair (MMR) genes (*MSH2*, *MSH6*, *MLH1*, and *PMS2)* are associated with microsatellite instability and a hypermutator phenotype in numerous systemic cancers, and germline MMR mutations have been implicated in multi-organ tumor syndromes. In gliomas, MMR mutations can function as an adaptive response to alkylating chemotherapy, although there are well-documented cases of germline and sporadic mutations, with detrimental effects on patient survival.

**Methods:**

The clinical, pathologic, and molecular features of 18 IDH-mutant astrocytomas and 20 IDH-wild-type glioblastomas with MMR mutations in the primary tumor were analyzed in comparison to 361 IDH-mutant and 906 IDH-wild-type tumors without MMR mutations. In addition, 12 IDH-mutant astrocytomas and 18 IDH-wild-type glioblastomas that developed MMR mutations between initial presentation and tumor recurrence were analyzed in comparison to 50 IDH-mutant and 104 IDH-wild-type cases that remained MMR-wild-type at recurrence.

**Results:**

In both IDH-mutant astrocytoma and IDH-wild-type glioblastoma cohorts, the presence of MMR mutation in primary tumors was associated with significantly higher tumor mutation burden (TMB) (*P* < .0001); however, MMR mutations only resulted in worse overall survival in the IDH-mutant astrocytomas (*P* = .0069). In addition, gain of MMR mutation between the primary and recurrent surgical specimen occurred more frequently with temozolomide therapy (*P* = .0073), and resulted in a substantial increase in TMB (*P* < .0001), higher grade (*P* = .0119), and worse post-recurrence survival (*P* = .0022) in the IDH-mutant astrocytoma cohort.

**Conclusions:**

These results suggest that whether present initially or in response to therapy, MMR mutations significantly affect TMB but appear to only influence the clinical outcome in IDH-mutant astrocytoma subsets.

**Key Points**

- Mismatch repair (MMR) gene mutations may be inherited, sporadic, or arise in response to therapy in astrocytomas and glioblastomas.
- MMR mutations increase tumor mutation burden in IDH-mutant astrocytomas and IDH-wild-type glioblastomas, but only impair survival in astrocytomas.

**Importance of the Study**

IDH-mutant astrocytomas are considered to be a distinct entity from IDH-wild-type glioblastoma with different underlying biology and better patient outcomes. Within each of these glioma subtypes however, there is heterogeneity of outcome that is only beginning to be understood in relation to the molecular drivers, intercellular molecular heterogeneity, and variable response to therapy. Herein, we evaluate the effect of mutations in mismatch repair (MMR) genes (*MSH2*, *MSH6*, *MLH1*, and *PMS2*), occurring both in the initial tumor and in subsequent recurrences of previously MMR-wild-type IDH-mutant astrocytomas and IDH-wild-type glioblastomas. These data demonstrate that MMR mutations lead to a significant increase in tumor mutation burden in both tumor types, but only affect patient survival in IDH-mutant astrocytomas. These findings suggest a potential source of heterogeneity in clinical outcomes amongst some IDH-mutant astrocytomas that have the potential to be routinely assessed in gliomas at the time of diagnosis.

Diffuse gliomas are the second most common primary intracranial neoplasm in adults, but the most common malignant central nervous system (CNS) tumor type. With over 16 000 cases occurring annually in the United States (19.3% of CNS tumors),^1^ diffuse gliomas are currently segregated into three major subgroups based on combined histologic and molecular criteria.^2^ Oligodendrogliomas are currently defined based on the presence of mutation in either *IDH1* or *IDH2* as well as codeletion of chromosomal arms 1p and 19q, IDH-mutant astrocytomas are defined as diffuse gliomas with *IDH1* or *IDH2* mutation in the absence of 1p/19q codeletion (and typically mutation in *TP53* and/or *ATRX*), and IDH-wild-type glioblastomas are defined as diffuse gliomas in the absence of mutation in *IDH1* or *IDH2*.^2,3^

Since the importance of these initial molecular features was first recognized, many studies have demonstrated additional molecular prognostic markers, with *EGFR* amplification, *TERT* promoter mutation, and/or simultaneous gain of chromosome 7 and loss of chromosome 10 (+7/−10) and homozygous deletion of *CDKN2A* being codified as the equivalent of grade 4 histologic features in IDH-wild-type glioblastoma and IDH-mutant astrocytomas, respectively.^2,4,5^ However, there remains heterogeneity of clinical outcome within both IDH-mutant and IDH-wild-type groups that is not fully addressed by the current diagnostic and prognostic criteria, but may be in part explained by the presence of additional genetic and epigenetic alterations in subpopulations of these glioma categories.^6–13^ One such alteration may be the presence of mutations in DNA mismatch repair (MMR) proteins, including *MSH2*, *MSH6*, *MLH1*, and *PMS2*.

Mutations in mismatch repair (MMR) genes have been shown to occur in diffuse gliomas as a consequence of alkylating chemotherapy with temozolomide (TMZ),^14–16^ and treatment with alkylating agents has been shown to induce a hypermutator phenotype, particularly in IDH-mutant astrocytomas, where it occurred in 47% of treated IDH-mutant astrocytomas compared with 16% of IDH-wild-type glioblastomas.^16^ Hereditary mismatch repair deficiency-related conditions, including Lynch syndrome and constitutional mismatch repair deficiency syndrome (CMMRD), have also been associated with an increased risk of gliomas and other brain tumors, and recent studies have demonstrated a potential new entity dubbed “primary mismatch repair deficient IDH-mutant astrocytoma (PMMRDIA),” a group of IDH-mutant astrocytomas arising in the presence of germline MMR mutation deficiency.^17–20^ These cases occurred almost exclusively in children and young adults with decreased rates of *MGMT* promoter methylation, a separate DNA methylation cluster from other IDH-mutant astrocytomas, and an extremely poor prognosis.^20^ Subsequently, other examples of both sporadic and inherited MMR-mutation-associated gliomas and other brain tumors have been identified.^21,22^

In this study, we analyzed 18 IDH-mutant astrocytomas and 20 IDH-wild-type glioblastomas with MMR mutations at initial presentation, compared to 361 IDH-mutant astrocytomas and 906 IDH-wild-type glioblastomas without MMR mutations from institutional and publicly available cohorts of diffuse gliomas to evaluate the clinical, pathologic, and molecular features, as well as the prognostic impact of MMR mutations in these tumors. We also analyzed an independent group of 12 IDH-mutant astrocytomas and 18 IDH-wild-type glioblastomas which were wild-type for MMR genes at the first resection but developed an MMR mutation at tumor recurrence. These cases were compared to 50 IDH-mutant astrocytomas and 104 IDH-wild-type glioblastomas that remained wild-type for all MMR genes at recurrence to determine the longitudinal effect of developing these mutations, particularly in the context of temozolomide therapy.

## Methods

### Histology, immunohistochemistry, and molecular profiling

**Figure 1.**
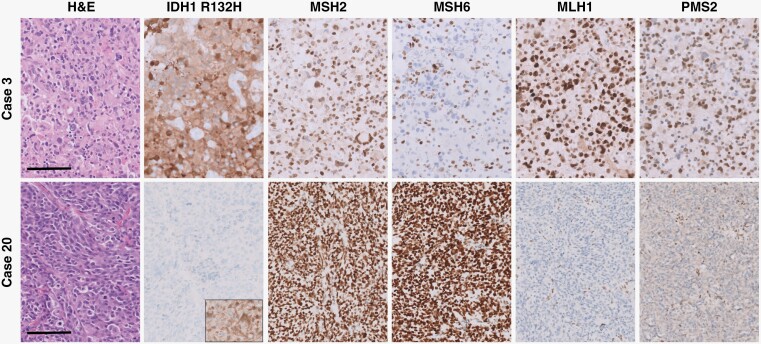
Histologic and immunohistochemical features of representative IDH-mutant astrocytoma and IDH-wild-type glioblastoma cases. There is loss of nuclear expression of MSH6 in tumor cells of the IDH-mutant case (case 3) and nuclear MLH1/PMS2 expression in tumor cells of the IDH-wild-type case (case 20), while background non-neoplastic cells have retained nuclear expression. All panels were taken at a total magnification of 200×. Scale bars = 100 µm.

Ten institutional cases of diffuse glioma with mutations in MMR proteins (*MSH2*, *MSH6*, *MLH1*, *and PMS2*) were identified, including 6 IDH-mutant astrocytomas and 4 IDH-wild-type glioblastomas. All tissue samples were obtained under approved Institutional Review Board protocols and appropriate consent for research. Hematoxylin and eosin (H&E) staining and immunohistochemistry (IHC) were performed on 4 µm-thick sections after heat-induced epitope retrieval using CC1 (Ventana, Tucson, AZ) using standard protocols. IHC stains included GFAP (Agilent, Santa Clara CA), OLIG2 (Sigma-Aldrich, St. Louis, MO, USA), IDH1 R132H (Dianova, Hamburg, Germany), p53 (Agilent), ATRX (Sigma-Aldrich, St. Louis, MO), Ki-67 (Agilent), MSH2 (Agilent), MSH6 (Agilent), PMS2 (Agilent), and MLH1 (Agilent) using Ventana UltraView Universal DAB Detection kits. Examples of immunohistochemical staining for IDH1 R132H and MMR proteins (MSH2, MSH6, PMS2, and MLH1) are shown in Figure 1.^23,24^ Clinically validated targeted next-generation sequencing was performed on all institutional cases at either FoundationOne Laboratories (https://www.foundationmedicine.com/test/foundationone-cdx) (Foundation Medicine, Inc., Cambridge, MA) or University of Pennsylvania (https://www.pennmedicine.org/departments-and-centers/center-for-personalized-diagnostics/gene-panels) at the time of initial diagnosis, as previously described.^10,25^

### Public Dataset Analysis

To identify similar cases in public datasets, we queried online databases^26–28^ to evaluate multiple large cohorts of primary diffuse gliomas and paired primary and recurrent gliomas.^16,26,29–35^ All cases were reclassified using *IDH1/2*, *ATRX*, *TP53*, and 1p/19q status according to the 2021 WHO Classification of CNS Tumors.^2,3^ IDH-mutant astrocytomas were graded based on histologic features and the presence/absence of homozygous *CDKN2A* deletion,^2,5^ while IDH-wild-type glioblastomas were graded based on the presence/absence of at least one of the following: *EGFR* amplification, *TERT* promoter mutation, and/or simultaneous gain of chromosome 7 and loss of chromosome 10 (+7/−10).^2,4^ From these publicly available datasets, 12 IDH-mutant astrocytomas and 16 IDH-wild-type glioblastomas with MMR gene mutations at initial presentation^26,32,35^ and an independent group of 12 IDH-mutant astrocytomas and 18 IDH-wild-type glioblastomas which developed MMR mutation between the initial specimen and tumor recurrence^16,32,35^ were identified and compared to 411 IDH-mutant, MMR-wild-type astrocytomas (50 with paired initial/recurrent specimen molecular analysis and 361 without paired analysis) and 1010 IDH-wild-type, MMR-wild-type glioblastomas (104 with paired initial/recurrent specimen molecular analysis and 906 without paired analysis). Gene expression data, mutation data, and copy number variation (CNV) data were downloaded and analyzed as previously described in detail.^10,36–39^ The variant allele frequency (VAF) for each mutation was defined as the ratio of mutant alleles divided by total alleles.^40,41^ The fraction of the genome with copy number alterations was calculated from the above data as the fraction of the genome with log2 of copy number > 0.3 following the procedure used in cBioPortal.^28^ Differential analysis and visualization of mutations were done using Maftools.^42^ Variant annotation was performed using COSMIC^43^ and ClinVar.^44^ MMR and driver mutations are described in Supplementary Table 1.

### Statistical Analysis

The significance of the proportion of cases between cohorts with specific molecular alterations as well as histologic grade and patient gender were calculated using Fisher’s Exact test. Differences in patient age, tumor mutation burden (TMB), and CNV were evaluated using student’s *t*-test. The significance of differences between Kaplan–Meier survival curves were calculated using the Mantel-Cox test (Log-rank test) and HRs were calculated using the log-rank test. All statistical calculations were performed with GraphPad Prism (GraphPad, La Jolla, CA).

## Results

### Clinical, Histologic, and Immunohistochemical Features of Diffuse Gliomas With Mismatch Repair Deficiency

**Figure 2.**
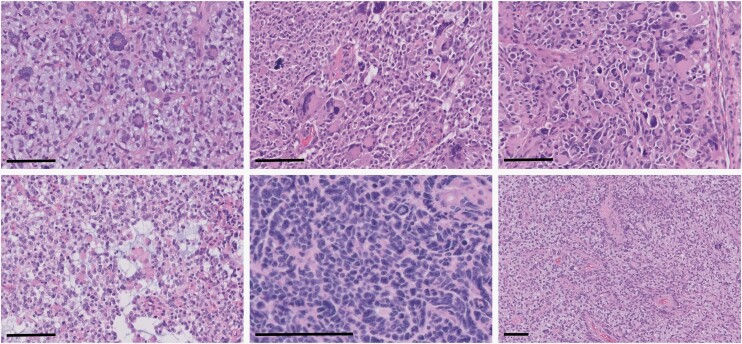
Representative examples of atypical histologic features identified in MMR-mutant diffuse gliomas. There were frequent multinucleated and bizarre giant cells (upper left and middle panels, total magnification 200×, scale bars = 100 µm), tripolar mitotic figures (upper right panel, total magnification 200×, scale bar = 100 µm), microcystic changes (bottom left panel, total magnification 200×, scale bar = 100 µm), primitive neuronal component (bottom middle panel, total magnification 400×, scale bar = 100 µm), and perivascular pseudorosettes (bottom right panel, total magnification 100×, scale bar = 100 µm).

No significant differences between patient gender or age at initial presentation were noted between IDH-mutant astrocytomas and IDH-wild-type glioblastomas with mutations in MMR genes compared to those without (Table 1). Institutional IDH-mutant astrocytomas and IDH-wild-type glioblastomas with mutations in MMR genes identified by targeted next-generation sequencing panels were subjected to a comprehensive histologic, immunohistochemical, and molecular workup. Each of these cases demonstrated loss of nuclear staining of at least one of the surveyed MMR proteins (MSH2, MSH6, MLH1, and PMS2), and all had evidence of retained nuclear staining of all MMR proteins in non-neoplastic tissue, including background glial cells, neurons, and endothelial cells (Figure 1). All institutional cases had conspicuous mitotic activity and either microvascular proliferation or tumor cell necrosis, consistent with WHO grade 4.^2^ All cases of IDH-mutant astrocytoma and IDH-wild-type glioblastoma demonstrated some form of atypical histologic features, including primitive neuronal features, giant cells with multinucleation and enlarged/bizarre nuclei, tripolar mitotic figures, microcystic areas, oligodendroglioma-like features, and perivascular pseudorosettes, reminiscent of ependymoma (Figure 2).^17,19,20^ DNA methylation profiling was available in three of the IDH-mutant astrocytomas and one IDH-wild-type glioblastoma; all were consistent with the integrated histologic/molecular diagnoses. No differences in GFAP, OLIG2, ATRX, p53, H3K27M, or H3K27me3 staining pattern, quantity, or intensity were noted in these MMR-deficient cases, and no differences in inflammatory response or imaging characteristics were identified.

**Table 1.** Clinical and Molecular Features in IDH-Mutant Astrocytomas and IDH-Wild-Type Glioblastomas With and Without MMR Gene Mutations in the Primary Tumor

Subgroup	*n*	Gender (M:F)	Age (Years)	2021 WHO Grade (2:3:4)	*CDKN2A* (Homozygous Deletion:Retained)^*^	*MGMT* Promoter (Methylated: Unmethylated)^*^	Tumor Mutation Burden (Mutations/Mb)	CNV (%)	Median PFS (Months)	Median OS (Months)
IDH-mutant astrocytoma										
MMR-mutant	18	10:8	35.1 ± 2.9	1:3:14	9:8	8:6	84.9 ± 13.0	21.3 ± 3.2	62.1	78.5
MMR-wild type	361	234:127	35.2 ± 0.5	155:118:88	67:294	93:60	15.1 ± 0.9	17.3 ± 0.7	97.8	161.0
*P*-value	-	=0.4548	=0.9658	**<0.0001**	**=0.0021**	=0.7829	**<0.0001**	=0.1381	=0.0528	**=0.0069**
IDH-wild-type glioblastoma										
MMR-mutant	20	9:11	51.7 ± 3.0	0:0:20	9:11	9:8	131.9 ± 14.8	20.6 ± 2.7	6.0	27.2
MMR-wild type	906	554:352	54.9 ± 0.5	0:0:906	256:261	158:343	13.6 ± 0.6	18.8 ± 0.3	10.0	22.0
*P*-value	-	=0.1670	=0.3707	-	=0.8207	=0.0702	**<0.0001**	=0.7457	=0.7789	=0.7373

**Note:** MMR, mismatch repair; CNV, copy number variation; PFS, progression-free survival; OS, overall survival; bold indicates statistical significance to a level of < 0.05;

^*^not all cases had available *CDKN2A* deletion or *MGMT* promoter methylation data.

### Molecular Features of IDH-Mutant Astrocytomas With Mismatch Repair Deficiency Present at Initial Diagnosis

**Figure 3.**
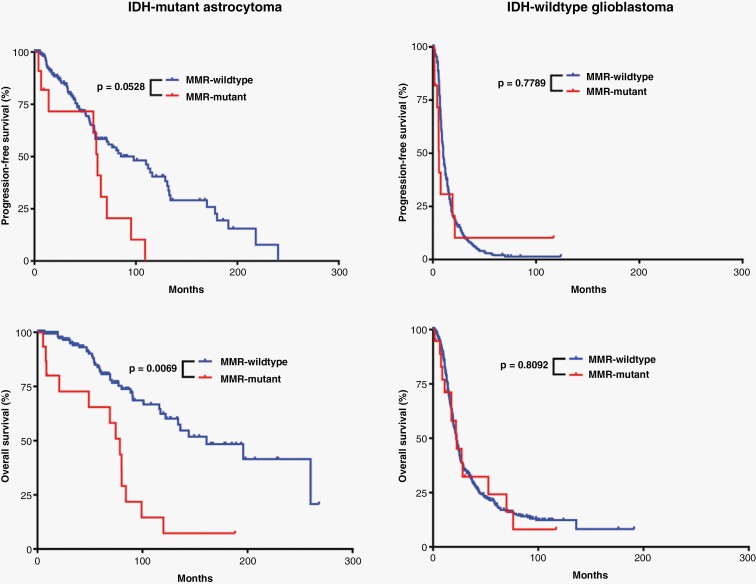
Effects of MMR mutations on glioma patient survival. Kaplan–Meier survival curves demonstrating a significant difference only in overall survival between IDH-mutant astrocytomas with and without MMR gene mutations present at initial biopsy/resection.

IDH-mutant astrocytomas with MMR mutations were significantly more likely to present at higher grade (*P* < .0001) and had more frequent homozygous *CDKN2A* deletion (*P* = .0021) than their MMR wild-type counterparts (Table 1). Compared to the MMR wild-type cases, the tumor mutation burden (TMB) in MMR-mutant cases was significantly higher (84.9 ± 13.0 vs. 15.1 ± 0.9; *P* < .0001) (Supplementary Figure 1), and these patients had shorter overall survival (median survival 78.5 months vs. 161 months; Hazard ratio (HR): 3.7 (1.4–10.0); *P* = .0069) but not recurrence/progression-free survival intervals, although there was a trend toward shorter recurrence-free interval in the MMR-mutant cases (*P* = .0528) (Figure 3). No differences were noted in the frequency of *MGMT* promoter methylation, level of genome-wide CNV, or other molecular features, including the frequency of *ATRX* mutation, *TP53* mutation, or *CDK4* amplification.

### Molecular Features of IDH-Wild-Type Glioblastomas With Mismatch Repair Deficiency Present at Initial Diagnosis

IDH-wild-type glioblastomas with MMR mutations had higher TMB compared to their MMR-wild-type counterparts (131.9 ± 14.8 vs. 13.6 ± 0.6; *P* < .0001) (Supplementary Figure 1). However, unlike the IDH-mutant cohort, this did not translate into any significant differences in patient survival (Figure 3). All tumors evaluated had at least one molecular feature consistent with glioblastoma (*EGFR* amplification, *TERT* promoter mutation, and/or + 7/−10).^2,4^ No differences were noted in the frequency of homozygous *CDKN2A* deletion or *MGMT* promoter methylation, CNV level, or other molecular features (Table 1).

### Molecular Analysis of Cases With Acquired Mismatch Repair Deficiency Occurring After Temozolomide Therapy

**Figure 4.**
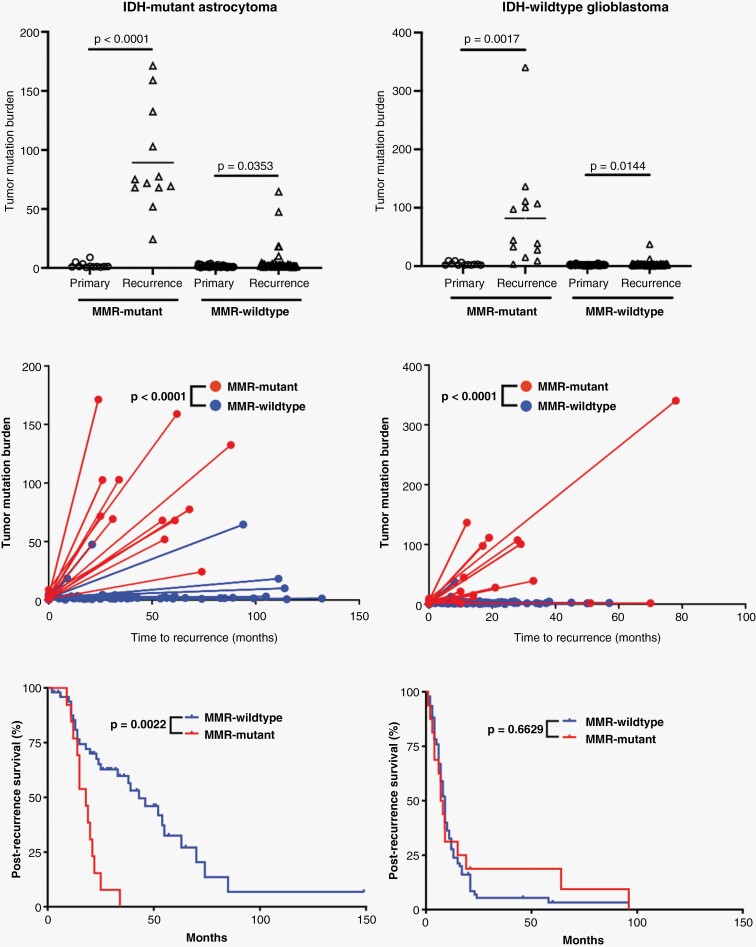
Effects of MMR mutations in response to alkylating agent therapy. Comparison of tumor mutation burden (TMB) in primary and recurrent tumors in IDH-mutant astrocytoma and IDH-wild-type glioblastoma cases where MMR gene mutations occurred between tumor samples and those without this feature. There is a significant increase in the TMB between primary and recurrent tumors in every category, and there is a significant difference in the level of TMB gains (ΔTMB) in MMR-mutant tumors compared to MMR-wild-type tumors in both IDH-mutant astrocytomas and IDH-wild-type glioblastomas. IDH-mutant astrocytomas that developed MMR gene mutations had significantly worse survival after recurrence compared to those without MMR genes, unlike in IDH-wild-type glioblastomas.

Both IDH-mutant and IDH-wild-type gliomas may acquire mutations in MMR genes and subsequent MMR protein loss as a protective response to alkylating agents, particularly temozolomide (TMZ).^14–16^ IDH-mutant astrocytomas that developed MMR gene mutations were significantly more likely to have been treated with TMZ compared to IDH-mutant astrocytomas which did not gain a mutation in MMR genes (*P* = .0073), but no significant difference was noted in IDH-wild-type glioblastomas with and without MMR mutation development (*P* = .2110) (Table 2). No significant differences were noted in terms of frequency of additional chemotherapies (*P* = .0909) or radiotherapy (*P* = .7307) in IDH-mutant astrocytomas with or without MMR mutation gain or frequency of additional chemotherapies (*P* = .0828) or radiotherapy (*P* = .9689) in IDH-wild-type glioblastomas. In IDH-mutant astrocytomas, the TMB was significantly elevated between the initial presentation and recurrence in both those with acquired MMR gene mutations (*P* < .0001) and those without (*P* = .0353) (Figure 4); however, the mean difference between TMB in primary tumors and recurrence (ΔTMB) differed significantly between these two groups. The ΔTMB in tumors which acquired an MMR mutation is 89.3 ± 12.5 compared to just 3.6 ± 1.6 in tumors which remained wild type for MMR genes (*P* < .0001). The mean variant allele frequency for MMR mutations occurring in the recurrent tumor specimens was significantly higher than the average VAF of all mutations that occurred exclusively in the recurrent tumors (*P* = .0053), suggesting that these MMR mutations occurred earlier than the majority of these additional mutations, although this may be affected by sampling, CNV, or the development of tumor subclones. IDH-mutant astrocytomas with these acquired MMR mutations had significantly shorter post-recurrence survival intervals compared to their MMR-wild-type counterparts (17.5 vs. 43.0 months; HR: 3.3 (1.2–8.8); *P* = .0022) (Figure 4 and Table 2). No difference was noted in 2021 WHO grade between MMR-mutant and MMR-wild-type IDH-mutant astrocytomas at either the initial tumor presentation or recurrence; however, 67% of cases with MMR mutations increased by at least one grade on recurrence (ΔGrade; 8/12), while only 28% of MMR-wild-type cases increased in grade on recurrence (14/50; *P* = .0119), suggesting that the development of an MMR mutation may in part drive progression to higher grade (Table 2). No additional significant differences in patient age, gender, or molecular features were noted between these two groups of IDH-mutant astrocytomas.

**Table 2.** Clinical and Molecular Features in Recurrent Gliomas With and Without MMR Gene Mutations in the Recurrence

Subgroup	*n*	Gender (M:F)	Age (years)	Initial 2021 WHO Grade (2:3:4)	Recurrence 2021 WHO Grade (2:3:4)	ΔGrade (% Increased at Recurrence)	*MGMT* Promoter^*^ (Methylated: Unmethylated)	TMZ Therapy^*^ (Treated Between Samples:Not Treated)	ΔTMB, Primary vs. Recurrence^*^ (Mutations/Mb)	Median Survival Post-Recurrence (Months)
IDH-mutant astrocytoma										
MMR-mutant	12	4:6	36.8 ± 3.0	9:3:0	4:1:7	67%	7:1	10:2	89.3 ± 12.5	17.5
MMR-wild type	50	35:15	34.7 ± 1.3	39:5:6	25:9:16	28%	22:4	16:28	3.6 ± 1.6	43.0
P-value	-	=0.1429	=0.4908	=0.2224	=0.3434	**=0.0119**	=0.8975	**=0.0073**	**<0.0001**	**=0.0022**
IDH-wild-type glioblastoma										
MMR-mutant	18	10:8	49.9 ± 2.5	0:0:18	0:0:18	-	7:2	16:0	62.7 ± 20.1	7.5
MMR-wild type	104	73:31	55.0 ± 1.1	0:0:104	0:0:104	-	23:49	80:11	3.1 ± 0.4	9.0
*P*-value	-	=0.2746	=0.0753	-	-	-	**=0.0111**	=0.2110	**<0.0001**	=0.6229

**Note:** MMR, mismatch repair; ΔGrade, change in grade between primary and recurrence, TMZ, temozolomide; ΔTMB, change in tumor mutation burden between primary and recrurrence; bold indicates statistical significance to a level of < 0.05;

^*^not all cases had available *MGMT* promoter methylation, TMZ therapy data, or paired TMB in primary and recurrent samples.

IDH-wild-type gliomas had significant elevation in TMB in both tumors which developed MMR gene mutations (*P* = .0017) and those that did not (*P* = .0144). Similar to IDH-mutant astrocytomas, the IDH-wild-type glioblastomas which acquired MMR mutations had significantly higher ΔTMB (62.7 ± 20.1) compared to those without MMR mutations in the recurrent specimen (3.1 ± 0.4; *P* < .0001) (Figure 4). The MMR-mutant cohort had more frequent MGMT promoter methylation (*P* = .0111), but there was no difference in post-recurrence survival rates between MMR-mutant and MMR-wild-type glioblastomas. No other differences in patient characteristics or molecular features were noted (Table 2).

## Discussion

There is wide-ranging literature on the function of DNA mismatch repair (MMR) genes as well as the impact of mutation or silencing of these genes in tumorigenesis.^45^ These genes function work in concert to form a base-pair mismatch recognition complex; the MSH2-MSH6 complex recognizes base-pair mismatches, then recruits MLH1-PMS2 to form a sliding clamp on the DNA strand, resulting in excision and replacement of the incorrect base-pair though POLE and POLD1 activity.^19,46^ Loss of function of any of these proteins results in microsatellite instability with a hypermutator phenotype and often a massive TMB, frequently leading to significant intercellular molecular heterogeneity, rapid tumor evolution, and selection of tumor clones with increased metastatic potential and resistance to therapies. This loss may occur via biallelic mutation, epigenetic silencing via CpG island methylation, loss of heterozygosity, or a combination of these mechanisms.^46^ Numerous previous studies have demonstrated that MMR mutations may also arise spontaneously or in response to alkylating therapy in existing IDH-mutant astrocytomas and IDH-wild-type glioblastomas,^14–16^ or may be present prior to tumor formation, particularly in cases with germline MMR mutation. Inherited MMR mutations are associated with Lynch syndrome/hereditary nonpolyposis colorectal cancer (HNPCC), a condition linked to GI, endometrial, and ovarian carcinomas, among more rare brain tumors. Other syndromes, such as constitutional mismatch repair deficiency (CMMRD), with inherited biallelic MMR gene mutations, present more commonly with various brain tumors, warranting a more rigorous and frequent CNS screening.^19^

A recently proposed entity, primary mismatch repair deficient IDH-mutant astrocytoma (PMMRDIA),^20^ is an IDH-mutant astrocytoma arising in the context of Lynch Syndrome or CMMRD with inherited mutations in *MSH2*, *MSH6*, or *MLH1*. These tumors occurred in children and young adults (median age 14 years old, range 9–54 years old), had a high frequency of hypermutant phenotype with microsatellite instability, increased rates of atypical morphology including giant cells and primitive neuronal features, and a low rate of *MGMT* promoter methylation. These patients also had extremely poor clinical outcomes with median survival of only 15 months, suggestive of grade 4 behavior regardless of other histologic or molecular features. While insufficient molecular data in non-neoplastic tissue was available in the present study to determine if the patients with MMR-mutant tumors at initial presentation had sporadic or germline MMR mutations, the patients in the current study presented at a much later age (median age 37 years old, youngest patient = 22 years old) compared to previous studies of IDH-mutant astrocytomas arising in patients with known congenital MMR deficiency syndromes (*P* < .0001).^18,20^ This later age of onset was not statistically different from the age of onset in patients with IDH-mutant, MMR-wild-type astrocytomas (35.2 ± 0.5 years; *P* = .9658), which may suggest that these mutations are sporadic. In addition, no differences were observed between rates of *MGMT* promoter methylation in the current cohort of IDH-mutant astrocytomas, unlike PMMRDIA cases. In the cohort of institutional cases, non-neoplastic cells (reactive glial cells, neurons, and endothelial cells) demonstrated retained nuclear immunoreactivity for all evaluated MMR proteins (Figure 1), which suggests that the MMR mutations in these 10 cases may be sporadic/non-germline.^23,24^ This immunohistochemical comparison of neoplastic and non-neoplastic cells could not be performed in the publicly available cohorts, which represents a limitation in the present study. The present cohort of IDH-mutant astrocytomas with MMR mutations in the primary specimens also had significantly higher 2021 WHO grade at initial presentation than their MMR-wild-type counterparts (Table 1), and a significantly higher percentage of cases with mutations in MMR genes recurred at higher grade (Table 2), suggesting that MMR mutations may be driving cases toward higher histologic and molecular grade. Similar to the PMMRDIA cases, MMR mutations had a pronounced effect on the overall survival of patients with IDH-mutant astrocytomas in this study (Tables 1–2 and Figures 3–4). It is unclear from these data why MMR mutation had no significant effect on survival in patients with IDH-wild-type glioblastoma, although this may be due in part to the fact that IDH-wild-type glioblastoma patients have such a dismal prognosis that there is not much capacity for MMR mutation to worsen the outcome. There were not enough cases with available DNA methylation profiling data in either IDH-mutant or IDH-wild-type cohorts to make meaningful conclusions on methylation clustering.

Known pathogenic MMR gene mutations occur almost exclusively in cases with high overall TMB, and the majority of cases that developed MMR mutations between the initial biopsy/resection specimen and a second were accompanied by a large rise in TMB, while tumors that did not develop MMR mutations (whether treated or untreated with alkylating chemotherapy) generally did not develop high TMB (Figure 4 and Table 2). In IDH-mutant astrocytomas, the development of these MMR gene mutations was significantly correlated with TMZ therapy between the primary tumor and recurrent specimen, although it remains unclear why MMR mutation only occurred in a subset of treated cases. In addition, the VAF of MMR mutations was significantly higher than the average VAF of all mutations acquired between the primary tumor resection and recurrence. This finding suggests that the MMR mutations likely occurred before the increase in TMB and did not simply arise in the background of an otherwise unrelated hypermutator phenotype (ie, MMR mutations were not simply random mutations that occurred alongside the hundreds to thousands of other mutations and more likely were the cause, not consequence, of the increased TMB). Previous work has suggested that tumors that develop this hypermutant phenotype as a result of alkylating chemotherapy do not necessarily have a worse outcome than tumors that do not in terms of overall survival from the time of first diagnosis.^16^ However, we show that if survival is measured starting with the recurrence in which a MMR mutation was first identified, patients that develop MMR gene mutations have significantly shortened post-recurrence survival intervals (Figure 4), suggesting that the gain of an MMR gene mutation after TMZ administration and subsequent increased TMB has a deleterious effect on survival in patients with IDH-mutant astrocytomas regardless of when in tumor evolution it occurs. These data are important for treatment considerations in patients with IDH-mutant astrocytomas, as they indicate that temozolomide therapy can induce MMR mutation and result in worse outcomes in a subset of these patients, but also suggests a role for immune checkpoint inhibitors to inform potential treatment strategies, although there is disagreement on the efficacy of immunotherapy in the setting of hypermutant gliomas.^19,47–49^

Taken together, these findings suggest that whether congenital, sporadic, or arising in response to therapy, mutations in MMR genes significantly affect the overall TMB in both IDH-mutant astrocytomas and IDH-wild-type glioblastomas but may only influence clinical outcomes in IDH-mutant astrocytomas. This detrimental impact on survival in IDH-mutant astrocytomas appears to follow the development of an MMR mutation, and so can negatively affect survival if it occurs subsequent to alkylating therapy. MMR mutations rarely occur in gliomas, but are important to recognize when present, as this can have consequences for clinical trial design. In the context of previous literature surrounding molecular prognostic factors in diffuse glioma subsets and the impact of MMR mutations in gliomas and other brain and systemic tumors, this report adds to the body of evidence suggesting that these mutations should be routinely screened for in both primary and recurrent gliomas, particularly IDH-mutant astrocytomas, which can be performed with standard immunohistochemical panels and targeted next-generation sequencing platforms.

## Supplementary Material

vdad085_suppl_Supplementary_Data

vdad085_suppl_Supplementary_Figure_S1

vdad085_suppl_Supplementary_Table_S1

## Data Availability

Portions of the data presented in this manuscript were derived from https://www.cbioportal.org, https://www.cancer.gov/tcga, and http://www.cgga.org.cn/. Additional data is available from the corresponding authors upon request.
