# Supplementary material for: Mismatch repair protein mutations in isocitrate dehydrogenase (IDH)-mutant astrocytoma and IDH-wild-type glioblastoma
Source: Neurooncol Adv. 2023 Jul 12;5(1):vdad085. doi: 10.1093/noajnl/vdad085 (PMC10406418; doi:10.1093/noajnl/vdad085)
Supplement: vdad085_suppl_Supplementary_Data [file vdad085_suppl_supplementary_data.docx]

**Supplemental Figure 1.** Tumor mutation burden and copy number variation in cases with and without mismatch repair gene mutations. Comparison of tumor mutation burden (TMB) and global copy number variation (CNV), expressed here as a percentage of the total genome, in IDH-mutant astrocytoma and IDH-wildtype glioblastoma cases with MMR gene mutations at initial presentation and those without these mutations. Significant differences were identified between MMR-wildtype and MMR-mutant cases in both IDH-mutant astrocytomas (p<0.0001) and IDH-wildtype glioblastomas (p=0.0001). No significant differences in global CNV were identified.

**Supplemental Table 1.** Mutations in tumor driver and MMR genes.
