# Supplementary figures and images for: Mismatch repair protein mutations in isocitrate dehydrogenase (IDH)-mutant astrocytoma and IDH-wild-type glioblastoma
Source: Neurooncol Adv. 2023 Jul 12;5(1):vdad085. doi: 10.1093/noajnl/vdad085 (PMC10406418; doi:10.1093/noajnl/vdad085)

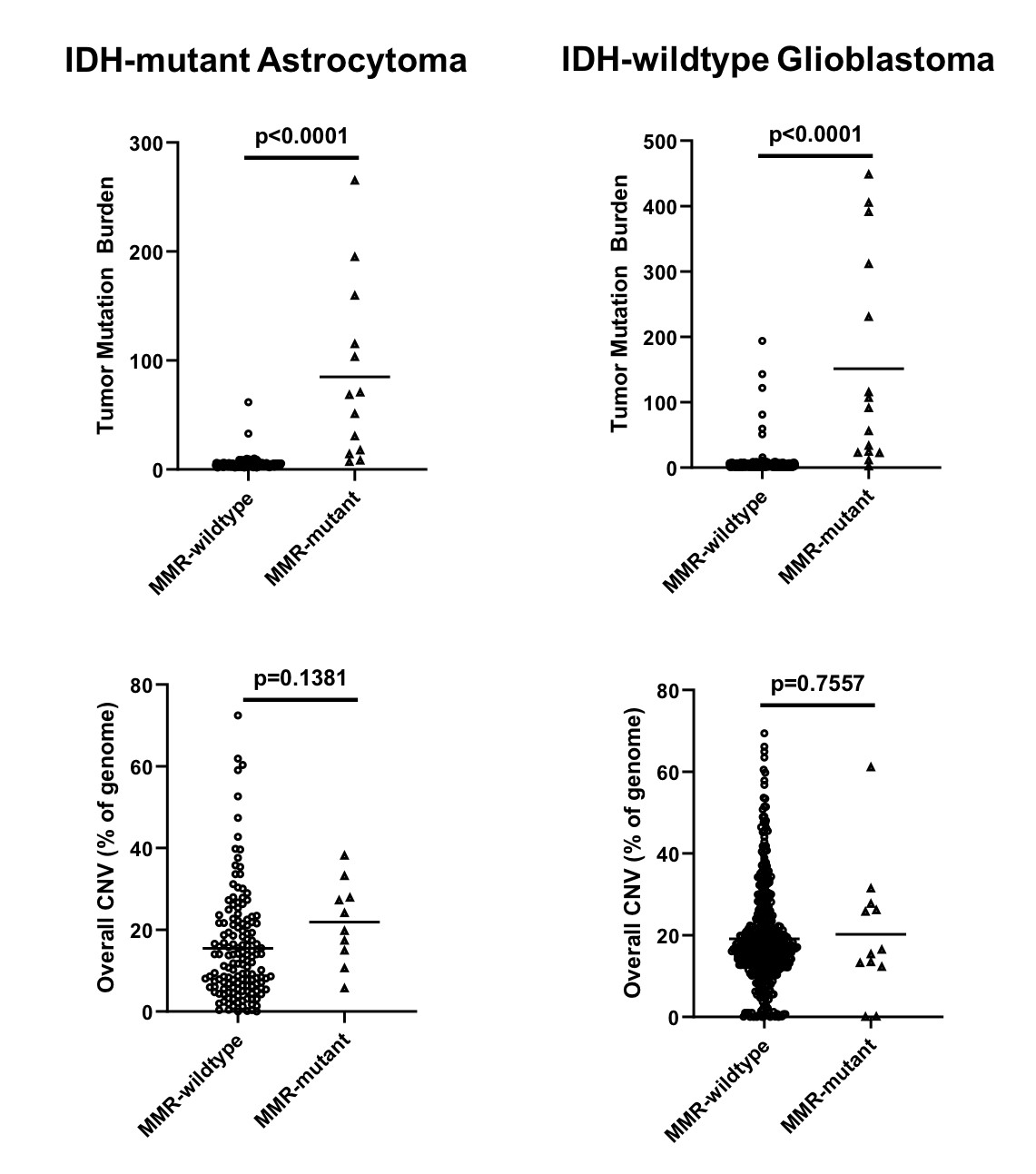

Supplement: vdad085_suppl_Supplementary_Figure_S1 [file vdad085_suppl_supplementary_figure_s1.jpeg]
