# Supplementary material for: Mismatch repair protein mutations in isocitrate dehydrogenase (IDH)-mutant astrocytoma and IDH-wild-type glioblastoma
Source: Neurooncol Adv. 2023 Jul 12;5(1):vdad085. doi: 10.1093/noajnl/vdad085 (PMC10406418; doi:10.1093/noajnl/vdad085)
Supplement: vdad085_suppl_Supplementary_Table_S1 [file vdad085_suppl_supplementary_table_s1.docx]

|  |  |  |  |  |  |  |  |  |  |  |
| --- | --- | --- | --- | --- | --- | --- | --- | --- | --- | --- |
|  | **Supplemental Table 1.** Mutations in tumor driver and MMR genes. | | | | | | | | |  |
|  |  | *IDH1/2* | *TP53* | *TERT* | *ATRX* | *MSH2* | *MSH6* | *MLH1* | *PMS2* |  |
|  | 1 | R132H (0.01) | R282W (0.90) | - | - | 1276+1G>A (0.87) | - | - | - |  |
|  | 2 | R132H (0.33) | R273C (0.40) | - | - | - | - | - | X55_splice (0.55) |  |
|  | 3 | R132H | Y236C | - | I360Rfs*6 | - | W105* | - | - |  |
|  | 4 | R132C | R273C | - | - | R524P | - | - | - |  |
|  | 5 | R132H (0.27) | D281H (0.48) | - | G249D (0.45) | - | - | A128P (0.36) | - |  |
|  | 6 | R132H | R248W | - | R1838* | - | T1219I | - | - |  |
|  | 7 | R132H (0.35) | R273C (0.71) | - | T887fs*19 (0.69) | - | - | R389Q (0.62) | - |  |
|  | 8 | R132H (0.45) | A138P (0.86) | - | E1395Dfs*95 (0.86) | - | X1147_splice (0.08) | - | - |  |
|  | 9 | R132H (0.30) | F109V (0.89) | - | V469Ifs*44 (0.36) | - | T1219I (0.42) | - | - |  |
|  | 10 | R132H (0.46) | H214Lfs*33 (0.97) | - | - | Q855* (0.19) | - | - | - |  |
|  | 11 | R132H (0.36) | R248Q (0.51) | - | I1049Nfs*4 (0.22) | - | - | X226_splice (0.46) | - |  |
|  | 12 | R132H (0.41) | R175H (0.81) | - | I1049Nfs*4 (0.60) | - | - | - | X236_splice (0.54) |  |
|  | 13 | R132H (0.48) | - | - | N428Yfs*5 (0.81) | - | T1219I (0.43) | - | - |  |
|  | 14 | R132C (0.37) | I195T (0.71) | - | T1582Nfs*19 (0.51) | - | T1219I (0.02) | - | - |  |
|  | 15 | R132H (0.10) | R273H (0.09) | - | I1049Nfs*4 (0.13) | - | T1219I (0.09) | - | - |  |
|  | 16 | R132H (0.10) | R175H (0.18) | - | T1582Nfs*19 (0.23) | - | - | X182_splice (0.23) | - |  |
|  | 17 | R132H | R273C | - | - | X71_splice | - | - | - |  |
|  | 18 | R132H (0.29) | R273C (0.79) | - | K1045* (0.34) | - | - | - | X55_splice (0.08) |  |
|  | 19 | - | C275Y | - | - | L187P | - | - | - |  |
|  | 20 | - | Y234C | - | - | - | - | X182_splice | - |  |
|  | 21 | - | E285K (0.73) | C250T (0.39) | - | - | - | R389Q (0.55) | - |  |
|  | 22 | - | X225_splice | - | - | - | - | R659* | - |  |
|  | 23 | - | R273H (0.47) | C228T (0.34) | - | Q413* (0.96) | - | - | - |  |
|  | 24 | - | X33_splice (0.37) | C250T (0.20) | - | - | - | T117M (0.37) | - |  |
|  | 25 | - | R175H (0.43) | - | - | - | - | X701_splice (0.35) | - |  |
|  | 26 | - | G226R (0.82) | C228T (0.34) | - | - | Q1048* (0.11) | - | - |  |
|  | 27 | - | G245S (0.38) | - | - | - | E544* (0.33) | - | - |  |
|  | 28 | - | R213* (0.39) | - | - | - | R248* (0.21) | - | - |  |
|  | 29 | - | R158H (0.26) | C250T (0.15) | - | X264_splice (0.26) | - | - | - |  |
|  | 30 | - | R158C (0.79) | - | - | X669_splice | - | - | - |  |
|  | 31 | - | - | C228T (0.27) | - | - | - | - | X55_splice (0.08) |  |
|  | 32 | - | - | C228T (0.28) | - | - | R240* (0.12) | - | - |  |
|  | 33 | - | - | C228T (0.27) | - | - | T1219I (0.30) | - | - |  |
|  | 34 | - | R273C (0.37) | - | - | X315_splice (0.66) | - | - | - |  |
|  | 35 | - | R306* (0.77) | - | - | - | E209* (0.27) | - | - |  |
|  | 36 | - | - | C228T (0.38) | - | X426_splice (0.39) | - | - | - |  |
|  | 37 | - | R248W (0.77) | C228T (0.40) | - | - | - | Q407* (0.40) | - |  |
|  | 38 | - | C275Y (0.52) | - | - | - | T1219I (0.24) | - | - |  |
|  | **Note:** when known, the variant allele frequency (VAF) is listed in parentheses after the specific mutation. | | | | | | | | |  |
|  |  |  |  |  |  |  |  |  |  |  |
